# Structure and Processing Properties of Nine Yam (*Dioscorea opposita* Thunb) Starches from South China: A Comparison Study

**DOI:** 10.3390/molecules27072254

**Published:** 2022-03-30

**Authors:** Jinhao Zou, Yan Li, Xiaojun Su, Feng Wang, Qingming Li, Huiping Xia

**Affiliations:** 1 Department of Food Science and Technology, College of Food Science and Technology, Hunan Agricultural University, No.1 Nongda Road, Furong District, Changsha 410128, China; lizjhy@163.com (J.Z.); 16674207524@189.cn (Y.L.); sxj@hunau.edu.cn (X.S.); wangfeng@hunau.edu.cn (F.W.); 2 Sericultural & Agri-Food Research Institute, Guangdong Academy of Agricultural Sciences, Key Laboratory of Functional Foods, Ministry of Agriculture and Rural Affairs, Guangdong Key Laboratory of Agricultural Products Processing, Guangzhou 510610, China

**Keywords:** Chinese yam starches, molecular structure, processing properties, physicochemical properties

## Abstract

In order to explore the processing and application potential of Chinese yam starch, nine kinds of Chinese yam starch (GY11, GY5, GY2, GXPY, LCY, SFY, MPY, SYPY, ASY) from South China were collected and characterized. The chemical composition, rheological properties, thermal properties, and in vitro starch digestion were compared, and the correlation between the structure and processing properties of these yam starches was analyzed using Pearson correlation. The results show that GY2 had the highest amylose content of 28.70%. All the yam starches were similarly elliptical, and all the yam starch gels showed pseudoplastic behavior. Yam starches showed similar pasting temperatures and resistant starch content, but SYPY showed the largest particle size (28.4 μm), SFY showed the highest setback (2712.33 cp), and LCY showed the highest peak viscosity (6145.67 cp) and breakdown (2672.33 cp). In addition, these yam starches also showed different crystal types (A-type, B-type, C-type), relative crystallinity (26.54–31.48%), the ratios of 1045/1022 cm^−1^ (0.836–1.213), pasting properties, and rheological properties, so the yam starches have different application potentials. The rheological and pasting properties were related to the structural properties of starch, such as DI, Mw, and particle size, and were also closely related to the thermodynamic properties. The appropriate processing methods and purposes of the processed products of these yam starches can be selected according to their characteristics.

## 1. Introduction

Yam (*Dioscorea opposita* Thunb), the dry tuber of *Dioscoreaceae* plants, is an important staple food crop in many tropical and subtropical countries [1]. In 2017, the global yam planted area reached 85.6535 million mu, making it the fourth-largest root crop after the planting area of potatoes, sweet potatoes, and cassava, according to the statistics of the Food and Agriculture Organization of the United Nations (FAO). In fact, yam is rich in nutrients and also contains a variety of biologically active substances, such as resistant starch, steroidal sapogenins, and mucilage polysaccharides. These biologically active substances have the effect of promoting health, but they have been neglected for a long time and have not been fully utilized [1].

Starch is the main nutrient component in yam (dry basis reaches 65.2–76.6%), and it can be used as a good source of carbohydrates in food [2]. The chemical composition (amylose content) [3], freeze–thaw stability [4], pasting properties [5], thermal properties [6], and rheological properties [7] of different yam starches grown in different regions are markedly different, and thus, yam starches have different application potentials in food and medicine [8,9]. For example, the amylopectin in yam starch has a high proportion of long chains, which can be used in crispy foods, salad dressings, and ready-made desserts [10]. Due to the high content of resistant starch (RS) in yam starch [11], it can be processed as a raw material or auxiliary material into potential functional foods [12,13]. In addition, researchers prepared an edible starch film by mixing yam starch [14] or modified yam starch [15] with glycerin, which can be used to preserve the freshness of fruits and meats.

Yam is a traditional Chinese edible and medicinal crop. There are many high-quality local yam varieties in South China, such as Mapu yam (geographical indication product), Lichuan yam (geographical indication product), and Guihuai yams (the cultivated yam varieties with high starch content are specially used for processing and can be planted in barren places). In recent years, the local planting scale of these yam varieties has become larger and larger, but because there is almost no research on processing, the lack of corresponding deep-processed products has caused great waste. Furthermore, previous research to date on the function, structure, and processing application potential of yam starch has been far less in-depth than that on common commercial tuber starches such as cassava, sweet potato, and potato [1]. This hinders the commercialization of yam starch. The difference in starch processing performance is due to differences in the structure of the starch itself [16,17,18]. Although there are currently some studies on the particle size, crystal structure, and molecular structure of yam starch, they have ignored the relationship between functional properties and the structure of yam starch. Therefore, comparing the functional properties of different varieties of yam starch and exploring the relationship between the structure and functional properties of yam starch is of great significance to the processing and application of yam starch and the breeding of yam varieties.

The purpose of this study was to provide useful information on yam starch application in food processing by measuring and comparing the structural and physicochemical properties of nine yam starches (GY11, GY5, GY2, GXPY, LCY, SFY, MPY, SYPY, ASY) selected from South China. Pearson correlation was used to analyze the relationship between the structure and properties of yam starch and to further understand the influence mechanism of starch structure on its properties so as to promote the development of this agricultural industry.

## 2. Results and Discussion

### 2.1. Chemical Composition and Molecular Features of Various Yam Starches

The chemical composition and molecular features of nine kinds of yam starch are shown in Table 1. There exist significant differences among them (*p* < 0.05). The moisture contents of the nine kinds of yam starch were less than 11%. The highest was for MPY starch (up to 10.11%), while the lowest was for ASY starch (5.86%). As shown in the table, the amylose content of these starches ranged from 4.83% (LCY starch) to 28.70% (GY2 starch). For most of these yam starches, the content was similar to that reported for 43 kinds of yam starches (15.08–27.07%) [19] and 10 kinds of yam starches (9.88–23.94%) [20] but slightly lower than that of *D. rotundata* and *D. alata* starch (30.26% and 31.02%) [19]. This may be related to the genetic factors of the plant, and it is also affected by the environmental temperature and light intensity. The amylose content is closely related to gelatinization properties, gel texture properties, and the processing properties of starch [3]. For example, for LCY, the variety with the lowest amylose content, the taste is soft and waxy. However, GY2, the variety with the highest amylose content, can be used for processing starch noodles [21]. The lipid (dry basis) content of each yam starch was below 0.5%, and the protein content was below 0.3%. The results indicate that the extracted starches were relatively clean and of high purity.

**Figure 1:**
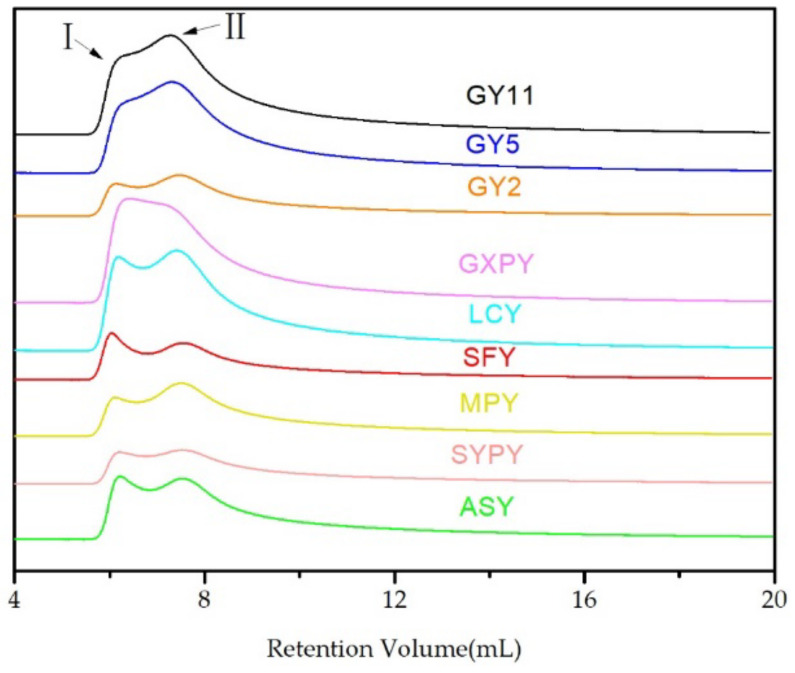
The gel permeation chromatogram of yam starch. Remark: I: Amylopectin elution peak; II: Amylose elution peak.

The molecular weight distribution of the nine kinds of yam starch is shown in Figure 1. The molecular weight distribution of each yam starch was composed of parts I and II (shown by arrows in the figure). Amylopectin has a higher molecular weight and was first eluted, followed by amylose with a lower molecular weight. Therefore, part I should be amylopectin, and part Ⅱ should be amylose. Table 1 shows the weight-average molecular weight (Mw), average radius of gyration (Rg), and dispersity index (DI) of the nine kinds of yam starch. The DI is the ratio of Mw to the number-average molecular weight (Mn). The closer the DI value is to 1, the more unitary the composition of the sample, while the larger the value, the more complex the composition and molecular weight distribution of the sample [22]. The Mw, Rg, and DI of the nine kinds of yam starches were in the ranges 4.416 × 10^7^–8.353 × 10^7^ g/mol, 178.255–228.601 nm, and 1.381–4.959, respectively. These values are much smaller than those of the 10 yam starches reported by Rolland (Mw, 1.88 × 10^8^–3.27 × 10^8^ g/mol; Rg, 261 nm–396 nm; DI, 8.82–21.66) [23]. Generally, a larger Rg value corresponds to a higher content or degree of branching of amylopectin [22,24]. Among the nine kinds of yam starch, SFY starch had the maximum Rg of 228.601 nm, while GY11 starch had the minimum Rg of 178.255 nm, which shows that SFY starch has a higher degree of branching of amylopectin. The DI of ASY starch was 4.959, and the DI of GXPY starch was 1.381, which was the closest to 1. This shows that ASY starch has the widest molecular weight distribution, and GXPY starch has the narrowest molecular weight distribution.

### 2.2. Scanning Electron Microscopy and Particle Size Distribution of Various Yam Starches

**Figure 2:**
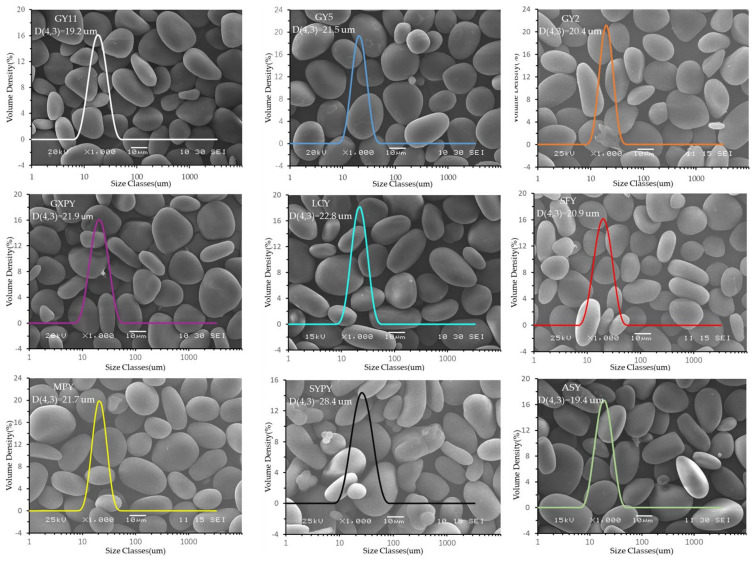
SEM micrographs and particle size distribution of yam starch.

The granular morphology of yam starches was observed with a scanning electron microscope. As presented in Figure 2, all yam starch granules exhibited an elliptical shape with a smooth surface containing few cracks and depressions, which is similar to many yam starch varieties [3,10,25]. The average particle size of the nine kinds of yam starch was measured with a laser particle size analyzer. The results show that the particles of each yam starch were relatively uniformly distributed with a single-peak distribution. They ranged from 19.2 μm (GY11) to 28.4 μm (SYPY). The average particle size of yam starch was affected by the variety and growth environment. According to reports, the average particle size of yam starch can be as low as 0.4 μm [7]. In addition, due to the drying method and the existence of protein, the average particle size of some yam starch composite particles was as high as 80 μm [26]. The average particle size of yam starches in this article was lower than that of 43 kinds of yam starches reported by Otegbayo (29.50–49.50 μm, except for Esuru funfun D. *dumetorum*) [19]. The particle size of starch has a great influence on its processing and application. In the processing of starch-based foods, suitable starch raw materials can be selected according to the particle size [19].

### 2.3. Fourier Transform Infrared Spectrum (FTIR) of Various Yam Starches

**Figure 3:**
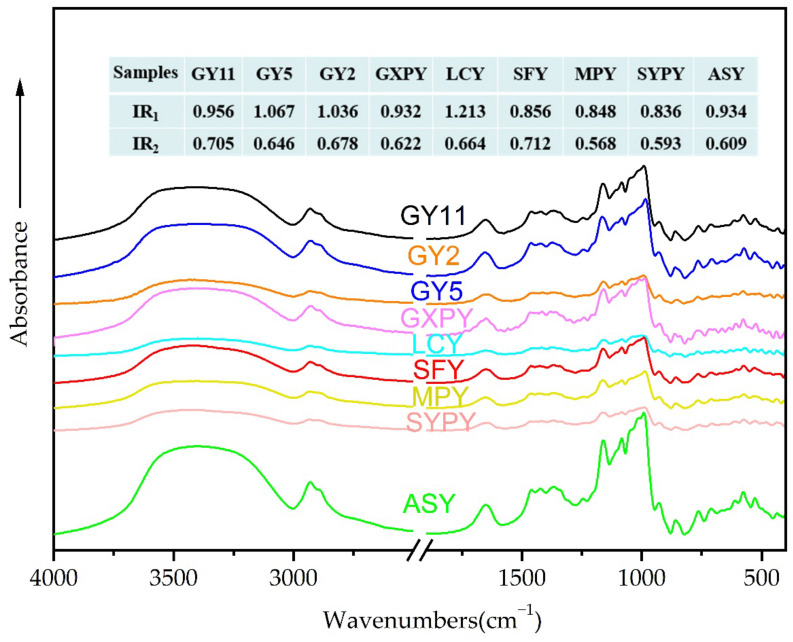
Fourier transform infrared (FTIR) of yam starch. Remark: IR_1_: the ratios of 1045/1022 cm^−1^; IR_2_: the ratios of 1022/995 cm^−1^.

FTIR spectroscopy has been a useful tool in monitoring structural changes in starches. Figure 3 shows the FTIR spectra of the nine kinds of yam starches. The FTIR waveforms of all yam starches were basically the same, and there were only differences in the position and intensity of the absorption peaks, which indicates that the types of functional groups possessed by the nine kinds of yam starch are similar, but there are differences in the amounts of functional groups. Fourier transform infrared was more sensitive to the conformation of the starch chain and the order of the helix. By deconvoluting the infrared spectrum of each yam starch, it is possible to quantitatively study the ratio of the ordered area to the amorphous area. The absorption peaks near 1045 cm^−1^ and 1022 cm^−1^ represent the ordered structure and amorphous structure of starch, respectively. The absorption peaks near 995 cm^−1^ were attributed to the bending vibration of C-OH, and the ratio of peak intensities of 1045/1022 cm^−1^ and 1022/995 cm^−1^ is an indicator of the ordered structure of starch, where the ratio of 1045/1022 cm^−1^ reflects the degree of order of starch. The larger the ratio, the higher the degree of order [3,10]. The results in Figure 3 indicate that the ratios of the ordered structure and amorphous structure of the nine kinds of yam starch are quite different. The maximum ratio of 1045/1022 cm^−1^ for LCY starch was 1.213, and the minimum ratio of 1045/1022 cm^−1^ for SYPY starch was 0.836. The maximum ratio of 1022/995 cm^−1^ for SFY starch was 0.712, and the minimum ratio of 1022/995 cm^−1^ for MPY starch was 0.568. The ratios of 1045/1022 cm^−1^ of the nine kinds of yam starches were larger than the ratios of 1022/995 cm^−1^, indicating that yam starch has a higher degree of order. This is different from the results reporting that the ratios of 1045/1022 cm^−1^ and 1022/995 cm^−1^ of seven kinds of purple sweet potato starches varied in the ranges of 0.689–0.887 and 0.850–0.974, respectively [18]. The ratios of 1045/1022 cm^−1^ and 1022/995 cm^−1^ of three kinds of Chinese yam starches varied in the ranges of 0.82–0.88 and 1.10–1.14, respectively [3]. This shows that the nine kinds of yam starches studied in this paper have a more orderly short-range ordered structure.

### 2.4. X-ray Diffraction Spectra of Various Yam Starches (XRD)

**Figure 4:**
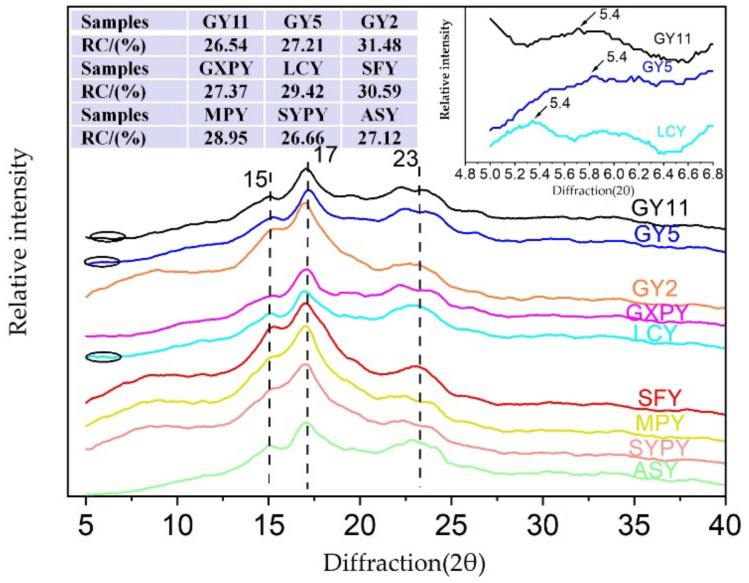
The X-ray diffraction spectra (XRD) of yam starch. Remark: RC: relative crystallinity.

The crystal type of starch can be classified into A, B, and C types according to the position and number of diffraction peaks in X-ray diffraction (XRD) spectra. As shown in Figure 4, the XRD spectra of SFY starch and ASY starch have pronounced single and strong peaks near 15°, 17°, and 23° (2θ), which suggests the typical A-type crystalline pattern. The XRD spectra of GY2, GXPY, MPY, and SYPY starch have strong diffraction peaks around 15° and 17° (2θ) and connected double peaks around 22° (2θ), which suggests that these yam starches show typical B-type crystalline diffractions. The XRD spectra of GY11 and GY5 starch have pronounced diffraction peaks near 15°, 17°, 22°, and 24° (2θ) and weak diffraction peaks near 5.6° (2θ). These were C-type crystalline starch, and the content of B-type crystals was relatively high, indicating C_B_-type starch. LCY has strong diffraction peaks near 15°, 17°, and 23° (2θ) and weaker diffraction peaks near 5.6° (2θ), which suggests that LCY starches are C-type. At 20° (2θ), there is an amorphous peak of amylose and lipids. Yam starches (GY11, GXPY, LCY, ASY) all have this peak, and their peak intensities are different. This reflects the difference between amylose content and lipid content in starches. Many studies have reported that different varieties of yam starches have pronounced diffraction peaks at 5.6° (2θ) [10,20,27]. However, six of the yam starches (GY2, GXPY, SFY, MPY, SYPY, ASY) reported in this article lack visible diffraction peaks at 5.6° (2θ). This indicates that the crystal structure of yam starch is affected by the yam variety.

The relative crystallinity (RC) of each yam starch is shown in Figure 4. It can be seen that the RC of yam starch ranged from 26.54% (GY11) to 31.48% (GY2). The RC of the nine kinds of yam starch in this article is similar to the results of the DH (29.16%), DM-1 (28.56%), D.CJ (28.12%), and D.PP (28.10%) yam starches reported by Jiang [20], which were higher than D.RC (20.54%), D.XM (19.48%), DM-2 (19.37%), D.TG (18.97%), and D.BY (12.02%) but much lower than DJ (51.68%). The result of RC measured by XRD was inconsistent with the ratio of 1045/1022 cm^−1^ measured by FTIR. FTIR mainly measures the ordered structure in short-range molecules, while XRD reflects the ordered structure in long-range molecules [28,29].

### 2.5. The Rheological Properties of Nine Kinds of Yam Starches

#### 2.5.1. Pasting Properties

The pasting characteristics of the nine kinds of yam starch are shown in Table 2. There were significant differences in the pasting characteristics of different varieties of yam starch (*p* < 0.05). The pasting temperature of the 9 kinds of yam starch ranged from 75.37 °C to 84.37 °C, which is higher than that of the 63 kinds of yam starches reported by Muluneh (66.7 °C–78.7 °C) [2] and the cassava starches from two locations reported by Tappiban (69.5 °C–75.3 °C) [30]. The breakdown value is the difference between the peak viscosity and the trough viscosity, and the setback is the difference between the final viscosity and the trough viscosity, the two respectively representing the hot paste stability and cold paste stability of starch paste. LCY starch and MPY starch had the highest breakdown value and lowest setback value, indicating that the viscosity of these two yam starches decreases quickly during the gelatinization process, and they could be used for foods with soft textures such as sweet dumplings and taro balls [31]. The SYPY starch had the lowest setback value, which means that it has the potential to become a raw material for weaning food [32]. The peak viscosity reflects the swelling ability of starch, and the final viscosity indicates the ability of the sample to form a sticky gel after pasting and cooling. The peak viscosity (6145.67 cp) of LCY starch was the largest, indicating that this starch has the strongest swelling ability. The trough viscosity (4687.67 cp) and final viscosity (6975.00 cp) of ASY starch were the largest, indicating that ASY starch has a strong ability to form paste and gel after pasting. The peak viscosity (2405.33 cp), trough viscosity (1247.67 cp), and final viscosity (1335.67 cp) of SYPY were the smallest, indicating that its swelling ability is weak, and it did not readily form a paste and form a stable gel after pasting. The peak viscosities of all yam starches in this article were greater than those of the five kinds of yam starches reported by Jayakody (starch concentration, 4% *w*/*v*) [33] and less than those of NSY (8590 cp) and HSY (7318 cp) reported by Shao (starch concentration, 14% *w*/*v*) [3]. This is not only related to the concentration of starch paste but also related to the particle size of starch and the amylopectin chain length [3,33,34].

#### 2.5.2. Dynamic Rheological Properties

**Figure 5:**
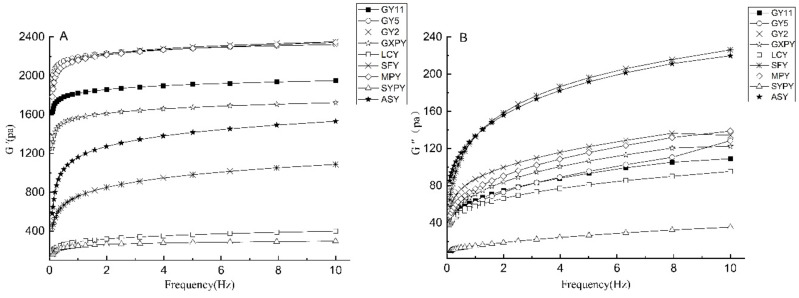

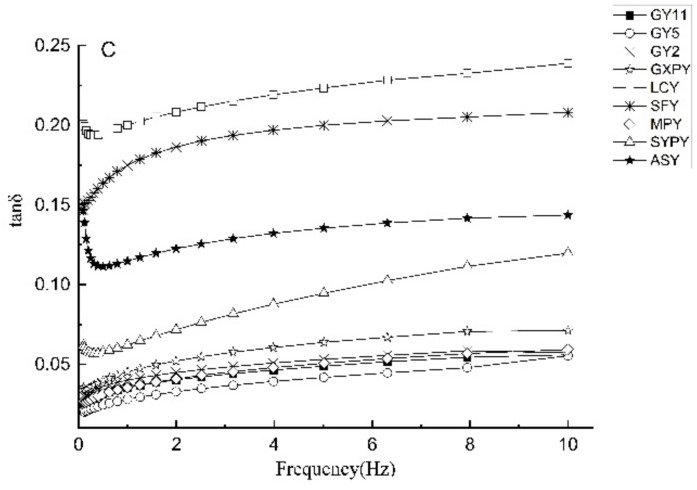
The elastic modulus (**A**), viscous modulus (**B**), and loss tangent angle (**C**) of yam starch. Remarks: G′: elastic modulus; G″: viscous modulus; tanδ: loss tangent angle.

Dynamic rheological properties can be used to determine the viscoelasticity of different samples, which is directly related to the actual processing characteristics and quality control of food. The elastic modulus (G′), viscous modulus G″, and loss tangent angle (tanδ) of the nine kinds of yam starch gels are shown in Figure 5. The G′ and G″ of the nine kinds of yam starch gels increased with the increase in frequency, and the tanδ of each yam starch gel was less than 1, which means that all the yam starch gels showed pseudoplastic behavior. The highest G’ value of GY2, MPY, and GY5 reached more than 2000 pa, while the G″ of each yam starch gel was less than 240 pa. The yam starch gels showed good gel elasticity and non-flowing properties, which means that the three kinds of yam starch may be used as a gelling agent and stabilizer to improve the rheological properties of set yogurt [8]. The rheological properties of starch gels are determined on the basis of the volume fraction, particle shape, and deformability of starch granules [6] and are also related to the molecular structure of starch [35].

### 2.6. The Thermal Properties of Nine Kinds of Yam Starches (DSC)

In the starch gelatinization process, the ordered crystalline structure is transformed into a disordered non-crystalline structure. This process is irreversible, and energy changes will occur. The differential scanning calorimetry (DSC) spectrum will show endothermic or exothermic peaks. Table 3 shows the results of the onset temperature (T_o_), peak temperature (T_p_), conclusion temperature (T_c_), and enthalpy of gelatinization (ΔH) of the nine kinds of yam starch. The T_o_, T_p_, T_c_, and ΔH of the nine kinds of yam starch were within 84.12–88.59 °C, 94.07–99.18 °C, 101.22–108.55 °C, and 23.08–31.23 J/g, respectively. In this study, the T_o_, T_p_, T_c_, and ΔH values of yam starch were higher than those reported by Wang for different varieties of yam starch [27]. This may be related to the crystallinity of the starch. Generally, the higher the degree of crystallinity, the neater the order of starch crystals, and the harder the gel formed by the starch [34]. LCY starch had the highest T_p_, T_c_, T_c_–T_o_, and ΔH, which is related to its low amylose content. Low amylose content means that starch has higher amylopectin content, so it is more difficult to gelatinize [25]. In addition, the thermal properties of starch are also affected by its particle size, amylopectin chain length, and other factors. In specific application areas, the raw materials can be selected according to the thermal properties of starch [27,36].

### 2.7. In Vitro Starch Digestion of Various Yam Starches

Starch digestibility is closely related to the diet and physiological function of the body, and it can have good prevention and control effects on the low starch digestibility associated with some chronic diseases. The digestibility results, including rapidly digested starch (RDS), slowly digested starch (SDS), and resistant starch (RS) of the nine kinds of yam starch, are shown in Table 4. There were significant differences in the digestibility of yam starch among different varieties (*p* < 0.05). The RS contents of the nine kinds of yam starch were significantly higher than SDS and RDS contents. The RS contents were above 90% among the nine kinds of starch; GXPY starch had the highest RS content of 97.43%, while the lowest was obtained for ASY starch (93.10%). The RS contents of 10 kinds of yam starch reported by Jiang were all above 70% [20]. Among them, D.J starch had the highest RS content of 90.80%, while the lowest was obtained for D.BY starch (76.50%). According to the report by Mighay [37], RS content values of different starches decreased in the order: yam (55.8%) > potato (48.5%) > arrowroot (17.5%) > taro (13.8%) > cassava (1.8%). It can be seen that yam starch is more resistant to digestion and has more beneficial physiological effects than other starches. Compared to the results of this study, the RS content of yam starch studied by Aprianita was low (13.20%) [38]. Generally, the digestibility of starch was related to the crystallinity of starch. The higher RS content of yam starch may be due to its higher crystallinity. In addition, the digestibility of starch may also be related to the size of starch granules, amylose content, and amylopectin branching degree [39,40,41]. The RDS contents and SDS contents of yam starches were low; the RDS contents of yam starches ranged from 0.19% (ASY) to 3.37% (GY11), and the SDS contents of yam starches ranged from 1.17% (GY11) to 6.71% (ASY). During the digestion process, the high crystallinity and tight molecular structure of starch will hinder the combination of amylolytic enzymes with starch molecules. The low digestibility (i.e., lower RDS contents and lower SDS contents) of the yam starches could be of significance to diabetics and the development of a healthy diet [7,42,43,44].

### 2.8. The Correlation between the Structures and Properties of Yam Starches

**Figure 6:**
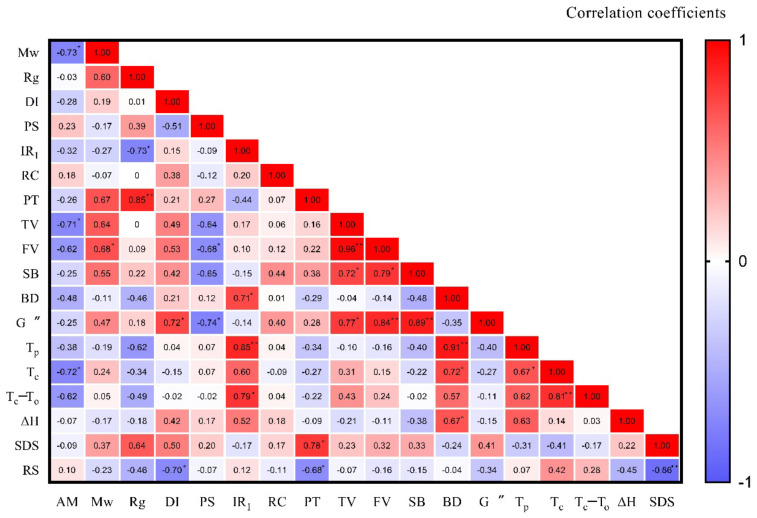
Pearson correlation coefficients between the structures and properties of yam starch. Remark: AM: amylose content; Mw: molecular weight; Rg: average radius of gyration; DI: dispersity index; PS: particle size; IR_1_: the ratios of 1045/1022 cm^−1^; RC: relative crystallinity; PT, pasting temperature; TV, final viscosity; FV, final viscosity; SB, setback; BD, break down; G″: viscous modulus; T_p_: peak temperature; T_c_: conclusion temperature; T_c_−T_o_:; ΔH: enthalpy of gelatinization;SDS: slowly digested starch. * and ** indicate significant differences at *p* < 0.05 and *p* < 0.01 levels, respectively.

Pearson correlation was used to analyze the relationship between the structural and physicochemical properties of the nine kinds of yam starches. As shown in Figure 6, the results show that amylose content was negatively correlated with Mw (r = −0.73, *p* < 0.05), TV (r = −0.71, *p* < 0.05), and T_c_ (r = −0.72, *p* < 0.05), which indicates that the higher the amylose content, the lower the Mw, TV, and Tc of yam starch. Rg is an indirect measurement of the degree of starch branching [24]. Rg was negatively correlated with IR_1_ (r = −0.73, *p* < 0.05) but positively correlated with PT (r = 0.85, *p* < 0.01), which indicates that the larger the Rg, the greater the limitation on the short-range order structure and the more difficult the starch gelatinization. IR1 was positively correlated with BV (r = 0.71, *p* < 0.05), T_p_ (r = 0.85, *p* < 0.01), and T_c_-T_o_ (r = 0.79, *p* < 0.05), which indicates that the short-range order structure is an important factor affecting the gelatinization and enthalpy change of starch. DI was negatively correlated with RS (r = −0.70, *p* < 0.05), and PT was negatively correlated with RS (r = −0.68, *p* < 0.05), which indicates that the smaller the DI and PT, the greater the RS content of starch.

The rheological properties reflect the industrial application potential of starch. The results show that DI was positively correlated with G″ (r = 0.72, *p* < 0.05). Mw was positively correlated with FV (r = 0.68, *p* < 0.05). Particle size was negatively correlated with FV (r = −0.68, *p* < 0.05) and G″ (r = −0.74, *p* < 0.05), which indicates that the rheological properties were related to the DI, Mw, and particle size of starch. The rheological properties and thermodynamic properties of starch were closely related. The results show that TV was positively correlated with FV (r = 0.96, *p* < 0.01), SB (r = 0.72, *p* < 0.05), and G″ (r = 0.77, *p* < 0.05). FV was positively correlated with SB (r = 0.79, *p* < 0.05) and G″ (r = 0.84, *p* < 0.01). SB was positively correlated with G″ (r = 0.89, *p* < 0.01). BV was positively correlated with T_p_ (r = 0.91, *p* < 0.01), T_c_ (r = 0.72, *p* < 0.05), and ΔH (r = 0.67, *p* < 0.05), which indicates that the G″ of starch was closely related to the viscosity change during the starch gelatinization process. The starch gelatinization process is also a process of starch enthalpy change. In this process, the starch granules expand and rupture, resulting in an increase in viscosity. T_p_, T_c_, and ΔH will affect the change in viscosity.

## 3. Materials and Methods

### 3.1. Materials

Mature tubers of 9 kinds of yams were from South China. Guilin yam No. 11 (GY11), Guilin yam No. 5 (GY5), Guilin yam No. 2 (GY2), and Guangxi purple yam (GXPY) were obtained from Nanning City, Guangxi Province. Lichuang yam (LCY) was obtained from Lichuan City, Hubei Province. Shuangfeng (SFY) was obtained from Loudi City, Hunan Province. Mapu yam (MPY) was obtained from Zhangzhou City, Fujian Province. Shaoyang purple yam (SYPY) was obtained from Shaoyang City, Hunan Province. Anshun yam (ASY) was obtained from Anshun City, Guizhou Province. The species of GY11, GY2, LCY, SFY, and ASY belong to *Dioscorea japonica* Thunb, and the species of GY5, GXPY, MPY, and SYPY belong to *Dioscorea alata* L. All chemical reagents used in the research were of analytical grade.

### 3.2. Starch Isolation

Starch was isolated using the wet-milling method previously described by Li [45]. In detail, the yams were peeled and then washed before being cut into 1–2 cm cubes. The freshly cut cubes were suspended in distilled water and then crushed in a grinder. The slurry was filtered through a 125 μm mesh screen. The material remaining on the sieve was rinsed twice with distilled water, with material that permeated the sieve being deposited in a bucket. After being deposited for 12 h, the water in the bucket was poured out, and distilled water was added to resuspend and deposit the bottom sediment. This process was repeated five times. The resultant starch was then collected and oven-dried for 48 h at 45 °C. The samples were ground and then sieved with a 150 μm mesh.

### 3.3. Chemical Composition

Amylose contents, protein contents, and lipid contents of the yam starches were determined in triplicate using the method described in AOAC (1990). The moisture contents of the yam starches were determined by oven drying and heating at 105 °C until constant weight [20].

### 3.4. Gel Permeation Chromatography (GPC)

The molecular weight of starch was determined using a gel permeation chromatography system (Viscotek TDA 305max, Malvern Instruments Ltd., Malvern, UK) [22]. In detail, 150 mg of starch was dissolved in 10 mL of 90% (*v*/*v*) dimethyl sulfoxide (DMSO) solution. Then, 1 mL of the suspension was removed and mixed with anhydrous methanol to precipitate the starch and then centrifuged at 4000 rpm/min for 20 min. The precipitate was re-dissolved in 5 mL of boiling water and then centrifuged at 4000 rpm/min for 30 min. The supernatant was injected into the chromatographic column (TGuard, T6000M, Malvern Instruments Ltd., Malvern, UK. The eluent was 50 mmol/L NaCl (containing 0.02% sodium azide), the elution rate was 0.5 mL/min, and the detectors were RI detector and small-angle light-scattering detector LALS (Viscotek SEC-MALS 20, Malvern Instruments Ltd., Malvern, UK). Based on this, the elution curve was drawn, and Origin 8.0 software was used to perform integral calculations.

### 3.5. Scanning Electron Microscope (SEM) and Particle Size

The dried starch sample was fixed on a conductive double-sided table and then sprayed with gold powder. The appearance of starch granules was observed and photographed with a scanning electron microscope (JSM-6380LV, Nippon Electronics Co., Ltd., Japan). The acceleration voltage was 25 kV during micrography. The size of starch particles was scanned using a laser particle sizer (Mastersizer 3000, Malvern Instruments Ltd., British) to determine the size distribution of the starch particles.

### 3.6. Fourier Transform Infrared Spectroscopy (FTIR)

Fourier transform infrared spectroscopy of starch was performed using the KBr tablet method previously described by Shao [3]. In detail, about 100 mg of KBr powder was mixed evenly with 1 mg of sample and then pressed into a tablet. It was scanned with an FTIR spectrometer (IRAffinity-1, Kyoto, Japan) in the range of 400–4000 cm^−1^, taking the wave number (cm^−1^) as the abscissa and the absorbance as the ordinate to obtain the infrared absorption spectrum.

### 3.7. X-ray Diffraction (XRD)

The crystal structure of the starch sample was measured with an X-ray diffractometer (XRD-6000, Shimadzu Instrument Co., Ltd., Japan) following the method described by Jiang [20]. In detail, the scanning mode was continuous. The step width was 0.02°. The Cu Kα radiation was 1.5406 Å at a voltage of 40 kV and current of 30 mA. The slit was 1.0°, 1.0°, and 0.3°. The scanning range of the diffraction angle (2θ) was 5–40° at a rate of 5°/min.

### 3.8. Rheological Properties

#### 3.8.1. Pasting Properties

Pasting properties of yam starches were determined using the method previously described by Jayakody [33]. In detail, an accurately weighed (3.00 ± 0.01 g) starch sample was dispersed into 25.0 mL of distilled water and then stirred evenly in a special rapid viscometer aluminum box of a rapid viscosity analyzer (RVA-S/N2112681, Swedish Perten Instrument Co., Ltd., Hägersten, Sweden). The temperature of RVA was kept at 50 °C for 1 min, then increased to 95 °C at 12 °C/min, kept at 95 °C for 2.5 min, decreased to 50 °C at 12 °C/min, and then kept at this temperature for 2 min.

#### 3.8.2. Dynamic Rheological Properties

Dynamic rheological properties of yam starches were determined using the method previously described by Zhu [35]. In detail, a 0.1 g/mL starch suspension was prepared, heated and gelatinized in a 95 °C water bath for 15 min after stirring, and then put into a rheometer (Kinexus pro+ rotational rheometer, Malvern Instruments Ltd., Malvern, UK). The excess sample outside the board was scraped off, and the test was started. The measuring platform of the rotational rheometer adopts a plate-plate measuring system; the plate diameter was 40 mm, and the plate spacing was 1 mm. The scanning frequency increased from 0.1 to 10 Hz at 25 °C.

### 3.9. Thermal Properties (DSC)

Thermal properties of yam starches were determined using a differential Scanning Calorimeter (DSC 3, Mettler Toledo, Greifensee, Switzerland) following the method previously described by Zhang [36]. In detail, a 30% (*w*/*w*) suspension of starch in water was prepared, sealed in a DSC crucible, and then kept at 25 ± 2 °C for 4 h to ensure that the starch samples were fully mixed with water. The empty crucible was used as a control. The samples were scanned from 20 °C to 200 °C at a rate of 10 °C/min under a nitrogen flow rate of 150 mL/min. The gelatinization enthalpy (∆H) and the onset (T_o_), peak (T_p_), and conclusion (T_c_) gelatinization temperatures of each sample were then determined from thermograms. All analyses were performed in duplicate, and mean values were calculated.

### 3.10. In Vitro Starch Digestion

In vitro starch digestion of yam starches was determined using the DNS method with reference to Amruthmahal [39]. In detail, yam starch (1 g), guar gum (50 mg) with sodium acetate buffer solution (20 mL, 0.1 M, pH 5.2), and 5 glass beads were incubated at 37 °C for 20 min. About 2000 u of porcine pancreatic α-amylase and glucoamylase (Beijing solarbio science & technology Co., Ltd., Beijing, China) was added, and the solution was shaken and hydrolyzed in a constant-temperature water bath (37 °C, 200 rpm/min). Then, 0.5 mL of the sample solution was removed at 20 min and 120 min, and 0.5 mL of absolute alcohol was added to inactivate the enzyme; the solution was centrifuged at 4000 rpm/min for 10 min, the supernatant was removed, and the DNS method was used to calculate the glucose content. G_20_ and G_120_ were calculated. The in vitro digestibility of starch was evaluated by three indicators: rapidly digested starch (RDS), slowly digested starch (SDS), and resistant starch (RS) (RDS refers to starch that is rapidly absorbed by the human small intestine within 20 min; SDS refers to starch that is digested and absorbed by the small intestine within 20 to 120 min; RS refers to starch that is not digested and absorbed by the small intestine within 120 min). The formulas are as follows:

RSD = (0.9 × G_20_ − 0.9 × FG) × 100%/TS (1)

SDS = (0.9 × G_120_ − 0.9 × G20) × 100%/TS (2)

RS = (0.9 × TG − 0.9 × FG) × 100%/TS − (RDS + SDS)(3)

Remark: G_20_ (mg) is the glucose released after 20 min of enzymatic hydrolysis. G_120_ (mg) is the glucose released after 120 min of enzymatic hydrolysis. FG (mg) is free glucose. TG (mg) is total glucose. TS (mg) is the dry basis weight of total starch, and 0.9 is the conversion factor.

### 3.11. Statistical Analysis

Excel 2010, Origin 8.0, and SPSS 20.0 were used for data processing and analysis, and the Duncan test method in the analysis of variance (ANOVA) was used for the significance analysis. The significance level of the test was *p* < 0.05, and all data are expressed as mean ± standard deviation (SD).

## 4. Conclusions

In this study, the structural and processing properties of nine kinds of yam starch were compared, and the correlation between the structure and properties of yam starch was explored. The results show that GY2 had the highest amylose content of 28.70%. All yam starches showed similar morphology (elliptical), and all the yam starch gels showed pseudoplastic behavior. However, these yam starches showed different crystal types (A-type, B-type, C-type). GY2 showed the highest relative crystallinity (31.48%), SFY showed the highest setback (2712.33 cp), and LCY showed the highest peak viscosity (6145.67 cp) and breakdown (2672.33 cp), but all yam starches showed similar pasting temperature and resistant starch content. The particle size and molecular structure of different varieties of yam starch were also different. From the results of Pearson correlation analysis, the higher the amylose content, the lower the Mw, TV, and Tc of yam starch. The larger the Rg, the greater the limitation on the short-range order structure and the greater the impact on starch gelatinization and enthalpy change. The rheological properties were related to the structural properties of starch, such as DI, Mw, and particle size, and were also closely related to the thermodynamic properties. Appropriate processing methods and products can be selected according to the different characteristics of different varieties of yam starch. This study provides useful information for the application of these yam starches in food.

## Figures and Tables

**Table 1:** Moisture content, amylose content, lipid content, protein content, and molecular features of yam starch.

Samples	Moisture (%)	AM (%)	Lipid Content (%)	Protein Content (%)	Mw (10^7^ g/mol)	Rg (nm)	DI
**GY11**	9.62 ± 0.05 ^b^	16.61 ± 0.49 ^c^	0.16 ± 0.02 ^e^	0.27 ± 0.01 ^b^	5.87 ± 0.02 ^g^	178.26 ± 0.05 ^i^	3.00 ± 0.03 ^e^
**GY5**	7.54 ± 0.02 ^e^	15.66 ± 0.32 ^d^	0.20 ± 0.01 ^c^	0.29 ± 0.00 ^a^	6.71 ± 0.08 ^e^	192.79 ± 0.09 ^f^	1.42 ± 0.06 ^h^
**GY2**	7.94 ± 0.05 ^d^	28.70 ± 0.89 ^a^	0.23 ± 0.02 ^b^	0.25 ± 0.00 ^c^	4.42 ± 0.09 ^h^	181.51 ± 0.04 ^h^	3.25 ± 0.08 ^d^
**GXPY**	9.28 ± 0.04 ^c^	11.38 ± 0.26 ^f^	0.18 ± 0.01 ^d^	0.20 ± 0.01 ^d^	7.18 ± 0.03 ^b^	200.31 ± 0.05 ^e^	1.38 ± 0.04 ^i^
**LCY**	7.21 ± 0.11 ^f^	4.83 ± 0.12 ^g^	0.14 ± 0.01 ^e^	0.10 ± 0.01 ^h^	6.67 ± 0.05 ^f^	186.55 ± 0.07 ^g^	3.87 ± 0.03 ^c^
**SFY**	10.01 ± 0.52 ^a^	13.16 ± 0.22 ^e^	0.48 ± 0.01 ^a^	0.14 ± 0.00 ^f^	8.35 ± 0.04 ^a^	228.60 ± 0.04 ^a^	4.31 ± 0.04 ^b^
**MPY**	10.11 ± 0.04 ^a^	15.56 ± 0.34 ^d^	0.03 ± 0.01 ^g^	0.07 ± 0.01 ^i^	6.99 ± 0.07 ^d^	213.01 ± 0.08 ^c^	2.04 ± 0.02 ^f^
**SYPY**	7.99 ± 0.10 ^d^	23.54. ± 0.94 ^b^	0.03 ± 0.00 ^g^	0.16 ± 0.01 ^e^	5.83 ± 0.07 ^g^	222.91 ± 0.07 ^b^	1.73 ± 0.05 ^g^
**ASY**	5.86 ± 0.05 ^g^	11.41 ± 0.26 ^f^	0.09 ± 0.00 ^f^	0.13 ± 0.00 ^g^	7.05 ± 0.03 ^c^	205.61 ± 0.04 ^d^	4.96 ± 0.04 ^a^

Remark: AM: amylose content; Mw: molecular weight; Rg: average radius of gyration; DI: dispersity index. Data (means ± SDs) in the same column with different letters are significantly different (*p* < 0.05).

**Table 2:** The pasting properties of yam starch.

Samples	PT/(℃)	PV/(cp)	TV/(cp)	FV/(cp)	SB/(cp)	BD/(cp)
**GY11**	75.37 ± 042 ^f^	4226.67 ± 42.03 ^f^	2589.67 ± 32.19 ^g^	3655.00 ± 41.22 ^f^	1065.33 ± 32.62 ^e^	1637.00 ± 26.46 ^c^
**GY5**	79.85 ± 0.48 ^d^	4119.67 ± 28.36 ^g^	3204.33 ± 28.54 ^e^	5028.00 ± 18.52 ^d^	1823.67 ± 10.02 ^c^	915.33 ± 11.72 ^e^
**GY2**	75.32 ± 0.51 ^f^	4114.00 ± 41.39 ^g^	2453.33 ± 71.67 ^h^	3432.00 ± 38.43 ^g^	979.67 ± 37.07 ^e^	1661.67 ± 87.46 ^c^
**GXPY**	76.50 ± 0.48 ^e^	5034.00 ± 14.53 ^d^	3927.33 ± 29.96 ^b^	5413.00 ± 26.51 ^c^	1485.67 ± 22.90 ^d^	1106.67 ± 21.50 ^d^
**LCY**	79.50 ± 0.48 ^d^	6145.67 ± 14.84 ^a^	3473.33 ± 30.35 ^d^	4534.00 ± 105.67 ^e^	1060.67 ± 100.55 ^e^	2672.33 ± 15.57 ^a^
**SFY**	84.37 ± 0.06 ^a^	4679.33 ± 18.34 ^e^	3746.33 ± 7.02 ^c^	6458.67 ± 27.68 ^b^	2712.33 ± 20.84 ^a^	933.00 ± 24.27 ^e^
**MPY**	83.40 ± 0.05 ^b^	5194.67 ± 76.96 ^c^	2985.67 ± 25.72 ^f^	3711.00 ± 26.23 ^f^	725.33 ± 12.86 ^f^	2209.00 ± 58.97 ^b^
**SYPY**	81.77 ± 0.06 ^c^	2405.33 ± 27.06 ^h^	1247.67 ± 24.19 ^i^	1335.67 ± 16.92 ^h^	88.00 ± 13.00 ^g^	1157.67 ± 36.23 ^d^
**ASY**	81.70 ± 0.00 ^c^	5558.00 ± 20.95 ^b^	4687.67 ± 10.21 ^a^	6975.00 ± 79.30 ^a^	2287.33 ± 86.64 ^b^	870.33 ± 22.74 ^e^

Remark: PT, pasting temperature; PV, peak viscosity; TV, final viscosity; FV, final viscosity; SB, setback; BD, break down;cp: viscosity unit. Data (means ± SDs) in the same column with different letters are significantly different (*p* < 0.05).

**Table 3:** The thermal properties of yam starch.

Samples	DSC				
	T_o_/(℃)	T_p_/(℃)	T_c_/(℃)	T_c_–T_o_/(℃)	ΔH/(J/g)
**GY11**	88.25 ± 1.28 ^a^	96.51 ± 2.74 ^ab^	103.42 ± 1.92 ^b^	15.17 ± 0.74 ^cd^	28.30 ± 0.96 ^b^
**GY5**	84.69 ± 0.64 ^b^	96.17 ± 2.76 ^b^	103.47 ± 3.84 ^b^	18.78 ± 4.44 ^b^	27.55 ± 1.25 ^b^
**GY2**	84.66 ± 0.63 ^b^	94.95 ± 1.05 ^bc^	101.22 ± 1.14 ^bc^	16.56 ± 0.97 ^bcd^	28.13 ± 0.39 ^b^
**GXPY**	87.68 ± 0.91 ^a^	94.37 ± 0.67 ^bc^	107.03 ± 1.19 ^a^	19.35 ± 0.27 ^b^	24.93 ± 0.80 ^cd^
**LCY**	85.20 ± 1.55 ^b^	99.18 ± 0.44 ^a^	108.55 ± 0.86 ^a^	23.35 ± 1.92 ^a^	31.23 ± 1.29 ^a^
**SFY**	88.59 ± 1.26 ^a^	94.16 ± 1.09 ^bc^	102.04 ± 2.11 ^bc^	13.44 ± 1.20 ^d^	29.09 ± 2.16 ^b^
**MPY**	85.28 ± 0.49 ^b^	94.07 ± 0.28 ^bc^	102.87 ± 0.69 ^b^	17.59 ± 1.12 ^bc^	23.08 ± 1.02 ^de^
**SYPY**	87.96 ± 1.15 ^a^	94.48 ± 2.11 ^bc^	101.61 ± 0.43 ^bc^	13.65 ± 0.74 ^d^	29.01 ± 1.67 ^b^
**ASY**	84.12 ± 0.51 ^b^	93.62 ± 2.08 ^bc^	101.79 ± 0.558 ^bc^	17.67 ± 0.78 ^bc^	26.79 ± 1.34 ^bc^

Remark: Data (means ± SDs) in the same column with different letters are significantly different (*p* < 0.05).

**Table 4:** The in vitro starch digestion of yam starch.

Samples	RDS/(%)	SDS/(%)	RS/(%)
**GY11**	3.37 ± 0.40 ^a^	1.17 ± 0.20 ^h^	95.46 ± 0.46 ^b^
**GY5**	0.25 ± 0.04 ^fg^	4.35 ± 0.23 ^d^	95.40 ± 0.22 ^b^
**GY2**	1.05 ± 0.19 ^c^	3.48 ± 0.24 ^f^	95.48 ± 0.32 ^b^
**GXPY**	0.70 ± 0.12 ^de^	1.87 ± 0.10 ^g^	97.43 ± 0.21 ^a^
**LCY**	1.47 ± 0.04 ^b^	4.24 ± 0.39 ^de^	94.28 ± 0.30 ^c^
**SFY**	0.50 ± 0.08 ^ef^	6.06 ± 0.50 ^b^	93.45 ± 0.55 ^de^
**MPY**	1.00 ± 0.08 ^c^	3.85 ± 0.05 ^ef^	95.15 ± 0.13 ^b^
**SYPY**	0.81 ± 0.12 ^cd^	5.30 ± 0.25 ^c^	93.90 ± 0.25 ^cd^
**ASY**	0.19 ± 0.03 ^g^	6.71 ± 0.20 ^a^	93.10 ± 0.18 ^e^

Remark: Data (means ± SDs) in the same column with different letters are significantly different (*p* < 0.05).

## Data Availability

Not applicable.

## Sample Availability

Samples of the compounds are not available from the authors.
